# Reply to C Pang and L Zhao

**DOI:** 10.1016/j.advnut.2026.100712

**Published:** 2026-09-04

**Authors:** Larissa L da Cruz, D Keith Williams, Sarah Sobik, Aline Andres

**Affiliations:** 1 Arkansas Children’s Nutrition Center, Little Rock, AR; 2 Department of Pediatrics, University of Arkansas for Medical Sciences, Little Rock, AR

Dear Editor:

Pang and Zhao emphasized their concerns regarding the claim made in the parent publication that the combined results presented in the review “support the safety of modern soy-based formula (SF) when used under appropriate clinical guidance.” The review presents current clinical guidance as a stepwise approach: breastfeeding is preferred; when possible, cow milk-based formula is recommended when breastfeeding is not possible, and SF is an alternative when cow milk-based formula is not tolerated. Pang and Zhao conclude by stating that “SF should remain restricted to infants with confirmed cow milk allergy who cannot tolerate hydrolyzed formula.” This conclusion aligns with our emphasis on use under appropriate clinical guidance. The review does not prove that SF is safe for every possible outcome over the entire life course. No study with follow-up to age 14 would be able to achieve that. What the review does suggest is that, among the outcomes measured in this prospective cohort, there were no observed major adverse patterns for children fed SF compared with cow milk-based formula. Overall, the study provides additional evidence for clinicians and families to make informed feeding decisions, recognizing that, in the absence of specific medical indications, parental preference often drives the formula choice.

In addition, Pang and Zhao cite concerns regarding “selective attrition” as an inherent generalizable limitation, which is clearly identified in the limitation section of the review and include details on the percent attrition “low rate of retention for the Beginnings Follow-Up Study (14 y-visit: 31% of enrolled participants, 36% eligible participants, and 49.4% of the completers at age 6).” Importantly, attrition in the beginnings study was consistent with other long-term pediatric cohort studies [[1], [2], [3]]. As noted in the review, the results should be interpreted within that context.

Pang and Zhao also state that “in nonrandomized designs, absence of evidence should be reported with its precision—confidence intervals and statistical power—rather than framed as evidence of absence.” Although we agree that confidence intervals and statistical power are important considerations when reporting findings from nonrandomized studies, the purpose of this review was to synthesize evidence collected over a 20-y period across multiple research domains. As a review article, we relied on and cited the original publications, where methodological details such as confidence intervals, power calculations, and measures of precision are reported.

Finally, Pang and Zhao appear to interpret several statements in the review more broadly than intended. They report that most outcomes were exploratory, that the conclusion “call for policy changes,” that “14 y is too short to assess true long-term safety,” and highlight outcomes such as “fertility, menstrual regularity, and adult metabolic phenotypes.” The Beginnings and Beginnings Follow-Up studies were designed to assess growth, body composition, and development from age 3 mo to 6 y and at age 14 y, and therefore, most presented outcomes were not exploratory; they were defined according to the study protocol based on the available scientific evidence. In addition, although reproductive outcomes such as adult fertility cannot be evaluated during adolescence, clinically relevant markers of pubertal status were assessed and did not differ among the feeding groups. The call for policy change should be carefully evaluated within the context of the full conclusion statement. “Collectively, these findings support the safety of modern SF when used under appropriate clinical guidance and reinforce the importance of optimizing infant nutrition policies, ensuring formula composition aligns with current scientific evidence and considers environmental and parental factors that influence infant long-term health. Within this context, the call for optimizing infant nutrition policies is framed outside the scope of the results presented in the review. Although the beginnings study does not establish the absolute lifelong safety of soy formula, it does provide substantial prospective evidence that, through adolescence, soy formula was not observed to have major adverse differences compared with cow milk-based formula across the outcomes examined. That interpretation is also consistent with supporting breastfeeding, when possible, cow milk-based formula when breastfeeding is not possible, and soy formula under appropriate clinical guidance when needed.

## Funding

Supported by USDA ARS Project # 6026-10700-001-000D.

## Conflict of interest

AA is an Editorial Board Member for Advances in Nutrition and played no role in the Journal's evaluation of the manuscript.
